# Investigation of Underlying Biological Association and Targets between Rejection of Renal Transplant and Renal Cancer

**DOI:** 10.1155/2023/5542233

**Published:** 2023-05-23

**Authors:** Yinwei Chen, Zhanpeng Liu, Qian Yu, Xu Sun, Shuai Wang, Qingyi Zhu, Jian Yang, Rongjiang Jiang

**Affiliations:** ^1^Department of Urology, The Second Affiliated Hospital of Nanjing Medical University, Nanjing, China; ^2^Department of Urology, The First Affiliated Hospital of Nanjing Medical University, Nanjing, China; ^3^College of Pediatrics, Nanjing Medical University, Nanjing, China; ^4^Department of Orthopedics, Huai'an No. 1 People's Hospital, Huai'an, China

## Abstract

**Background:**

Post-renal transplant patients have a high likelihood of developing renal cancer. However, the underlying biological mechanisms behind the development of renal cancer in post-kidney transplant patients remain to be elucidated. Therefore, this study aimed to investigate the underlying biological mechanism behind the development of renal cell carcinoma in post-renal transplant patients.

**Methods:**

Next-generation sequencing data and corresponding clinical information of patients with clear cell renal cell carcinoma (ccRCC) were obtained from The Cancer Genome Atlas Program (TCGA) database. The microarray data of kidney transplant patients with or without rejection response was obtained from the Gene Expression Omnibus (GEO) database. In addition, statistical analysis was conducted in R software.

**Results:**

We identified 55 upregulated genes in the transplant patients with rejection from the GEO datasets (GSE48581, GSE36059, and GSE98320). Furthermore, we conducted bioinformatics analyses, which showed that all of these genes were upregulated in ccRCC tissue. Moreover, a prognosis model was constructed based on four rejection-related genes, including *PLAC8*, *CSTA*, *AIM2*, and *LYZ*. The prognosis model showed excellent performance in prognosis prediction in a ccRCC cohort. In addition, the machine learning algorithms identified 19 rejection-related genes, including *PLAC8*, involved in ccRCC occurrence. Finally, the *PLAC8* was selected for further research, including its clinical and biological role.

**Conclusion:**

In all, our study provides novel insight into the transition from the rejection of renal transplant to renal cancer. Meanwhile, *PLAC8* could be a potential biomarker for ccRCC diagnosis and prognosis in post-kidney transplant patients.

## 1. Introduction

Renal cell carcinomas (RCCs) are malignant tumors originating in the urinary tubular epithelium and constitute about 80–90% of renal tumors [1]. RCC accounts for about 2–3% of malignant tumors in adults and 20% in children [2]. Among the Chinese, RCC is the second most commonly encountered genitourinary tumor after bladder tumors. In addition, RCC has a distant metastasis rate of about 15% at diagnosis. Furthermore, the average age of diagnosis for RCC is 64 years old. RCCs have a higher predominance in males than females, with a male-to-female ratio of 1.7 : 1. In addition, clear cell, chromophobe, and papillary RCCs of type I and II are the most common histological subtypes of RCCs [1]. Clear cell renal cell carcinoma (ccRCC) shows a distinct metabolic phenotype, particularly in patients with end-stage renal disease (ESRD), characterized by accumulation of sorbitol, a shift away from respiratory metabolism, and severe depletion of mitochondrial DNA [3].

Surgical resection is the primary treatment option for non-metastatic RCC, while medical treatment is the primary treatment option for metastatic RCC [4]. Due to progress in cancer research, more effective treatment options, including targeted and immune therapies, have become popular alternatives. However, the treatment options for RCC in patients with ESRD and kidney transplantation (Ktx) remain limited. Ktx is considered the standard treatment for ESRD [5].

Ktx is associated with a higher cancer incidence risk [6]. A previous meta-analysis study reported a 5- to 10-fold increase in the incidence of renal carcinoma after transplantation and a 0.3% incidence of renal carcinoma among patients with ESRD [7]. Furthermore, ESRD patients have a higher incidence of RCC than the general population. Moreover, the cancer incidence following Ktx ranges from 2% to 31%, depending on the type of cancer and follow-up period [6]. The increased cancer risk in kidney transplant patients is associated with immunosuppression [8]. Although the link between immunosuppression and tumorigenesis is not fully understood, a previous controlled trial reported that the intensity of immunosuppressive therapy following transplantation was associated with higher cancer risk [9]. A previous study reported a higher cancer-related mortality rate among 19,103 kidney transplant recipients with renal carcinoma accounting for 9.8% of all cancer-related deaths [10].

The rapid development of bioinformatics and the arrival of the big data era provide researchers with powerful tools [11–15]. In this study, we analyzed data gathered from The Cancer Genome Atlas Program (TCGA) database and the Gene Expression Omnibus (GEO) database to illustrate the underlying correlation between RCC and Ktx, as well as find the possible biological mechanism between them.

## 2. Methods

The whole flow chart of this study was shown in Figure S1.

### 2.1. Data Collection

Next-generation sequencing data and corresponding clinical information of patients with ccRCC were obtained from TCGA database. The baseline information of ccRCC patients enrolled in this study was shown in Table 1. For TCGA database, the original file of each patient was downloaded from TCGA-Genomic Data Commons in the “STAR-Counts” form. The author's own R code is used to extract the expression data of each patient (Transcripts Per Million (TPM) units) and integrate it into a gene expression matrix. The probe annotation was based on the human genomic reference file GRCh38.gtf. Before data analysis, the data was standardized and transformed into log2 (TPM + 1). The microarray data of kidney transplant patients with or without rejection response was obtained from the GEO database. For the GEO database, the GSE48581 (GPL570), GSE36059 (GPL570), and GSE98320 (GPL15207) datasets were selected to provide the transcriptional profile data for kidney transplant patients with or without rejection response. Data pre-processing was conducted before the data analysis: (i) probe annotation based on platform files; (ii) missing value completion; (iii) averaging the expression of duplicate genes. The baseline information of participants from GEO databases was shown in Table 2. Pan-cancer data was obtained from the USCS XENA website (https://xenabrowser.net/).

### 2.2. Differentially Expressed Genes Analysis

Differentially expressed genes (DEGs) analysis was utilized to identify the genes differentially expressed in two specific groups using the limma package under the set threshold value (|log2 FC| > 1 and *P* < 0.05) [16].

### 2.3. Protein Interaction Network

The search tool for the retrieval of interacting genes (STRING) was utilized to investigate the underlying protein interactions of these genes [17]. Detailed, the “meaning of network edges” = “evidence”; the “minimum required interaction score” = “medium confidence”. The Cytoscape software (version 3.7.2) was utilized for network visualization.

### 2.4. Biological Enrichment Analysis

The Gene Set Enrichment Analysis (GSEA) was used to illustrate the biological differences between two specific groups based on the Hallmark gene set [18]. The ClueGO, a Cytoscape software plug-in, was used to perform function enrichment and intuitive representation of input genes [19]. Furthermore, the Gene Ontology (GO) and Kyoto Encyclopedia of Genes and Genomes (KEGG) analyses were conducted using the clusterProfiler package.

### 2.5. Prognosis Analysis

Univariate Cox regression analysis was conducted to identify genes significantly associated with patients' prognosis at *P* < 0.05, followed by Least absolute shrinkage and selection operator (LASSO) regression analysis to identify the most significant variables. Furthermore, multivariate Cox regression analysis was conducted to identify the prognosis signature.

### 2.6. Machine Learning Algorithm

LASSO logistics regression and support vector machine - recursive feature elimination (SVM-RFE) algorithms were used to identify the characteristic variable between different groups [20]. LASSO regression is an adaptation of the popular linear regression algorithm. Through feature selection, LASSO removes redundant variables and reduces overfitting. Furthermore, SVM-RFE, another feature selection technique, can remove insignificant variables and screen relevant features, thus achieving a higher performance [21, 22]. Detailed, the “*n* fold” = “5”; the “halve.above” = “100”.

### 2.7. Statistical Analysis

All the analyses were conducted using the R software. The threshold statistical significance was set at 0.05. Different statistical methods are adopted according to different data distribution forms.

## 3. Results

### 3.1. Identification of DEGs Involved in Renal Transplant Rejection

**Figure 1:**
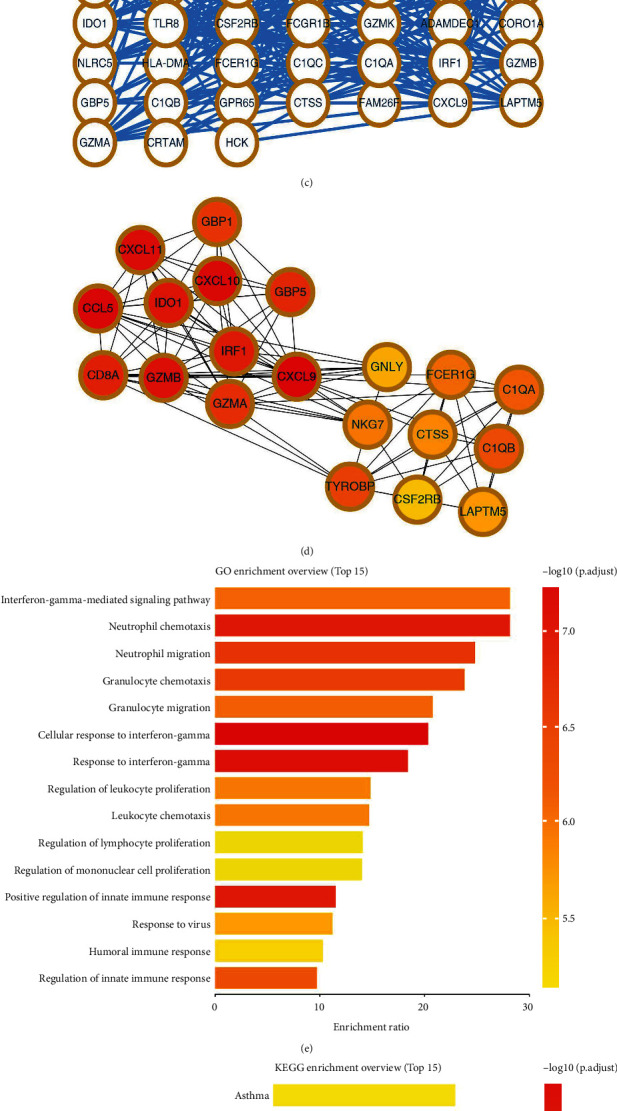
Identification of the genes involved in the rejection of the renal transplant. (a) The common genes upregulated in transplant patients with rejection intersected by GSE48581, GSE36059, and GSE98320; (b)the common genes downregulated in transplant patients with rejection intersected by GSE48581, GSE36059, and GSE98320; (c) PPI network of 55 upregulated genes; (d) the top 20 important nodes of PPI network; (e) GO analysis of the 55 upregulated genes; (f) KEGG analysis of 55 upregulated genes.

The transcription profile data for renal transplant patients with or without rejection was obtained from the GEO datasets (GSE48581, GSE36059, and GSE98320). We identified 55 upregulated genes in the transplant recipients that were rejection-related (Figures 1(a) and 1(b)). The protein protein interaction (PPI) network of these 55 genes is illustrated in Figure 1(c). The top 20 important genes identified in the PPI network were *GBP1*, *CXCL11*, *CCL5*, *CXCL10*, *IDO1*, *CD8A*, *GBP5*, *GZMB*, *IRF1*, *GZMA*, *CXCL9*, *GNLY*, *FCER1G*, *C1QA*, *NKG7*, *CTSS*, *C1QB*, *TYROBP*, *CSF2RB*, and *LAPTM5* (Figure 1(d)). The GO analysis showed that these 55 genes were mainly enriched in the interferon-gamma-medicated signaling pathway, neutrophil process, granulocyte process, and cellular response to interferon-gamma (Figure 1(e)). The KEGG analysis revealed that these genes were mainly involved in asthma, allograft rejection, graft versus host disease, and type I diabetes mellitus (Figure 1(f)).

### 3.2. Role of Rejection-Related Genes in Renal Cancers

**Figure 2:**
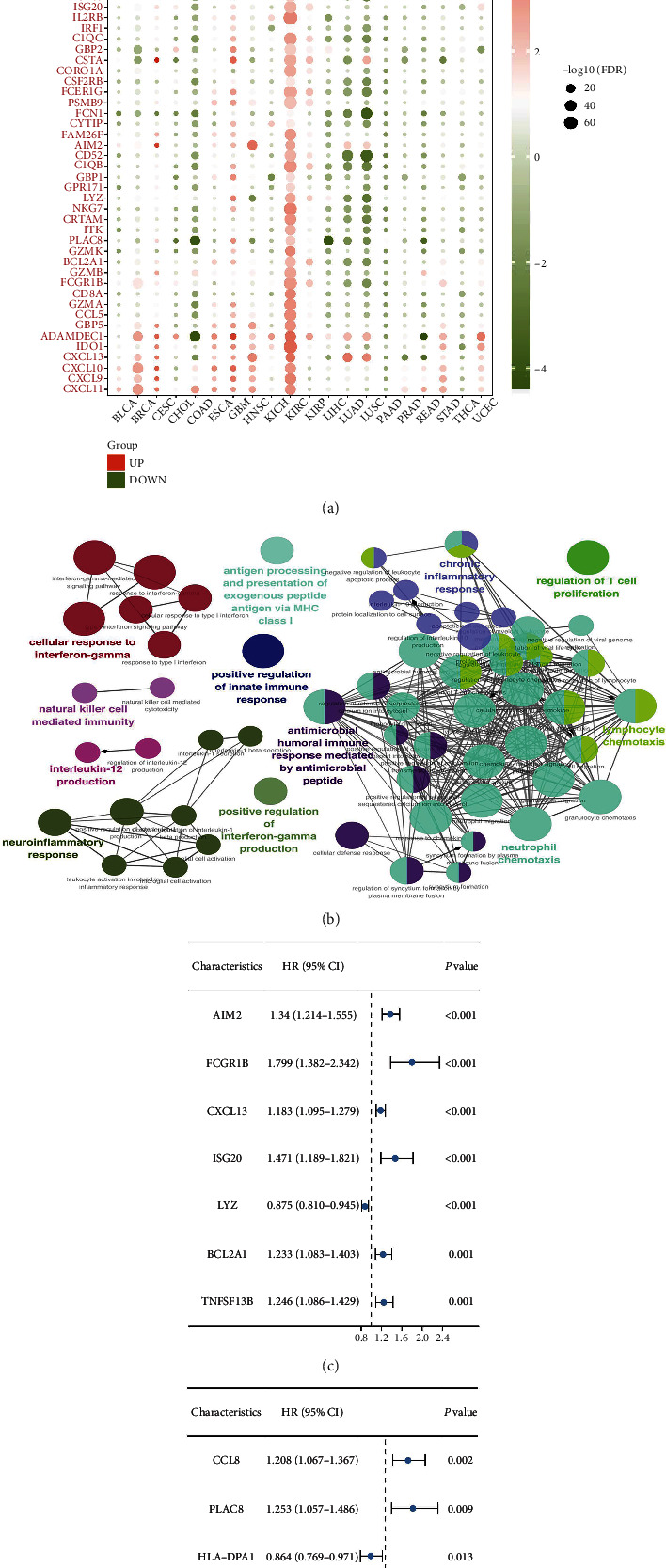
Expression pattern and prognosis analysis of 55 genes in TCGA database. (a) The expression pattern of the 55 upregulated genes in TCGA pan-cancer data; (b) ClueGO analysis of the 55 upregulated genes; (c) and (d) univariate Cox regression analysis of the 55 upregulated genes in ccRCC patients in TCGA.

**Figure 3:**
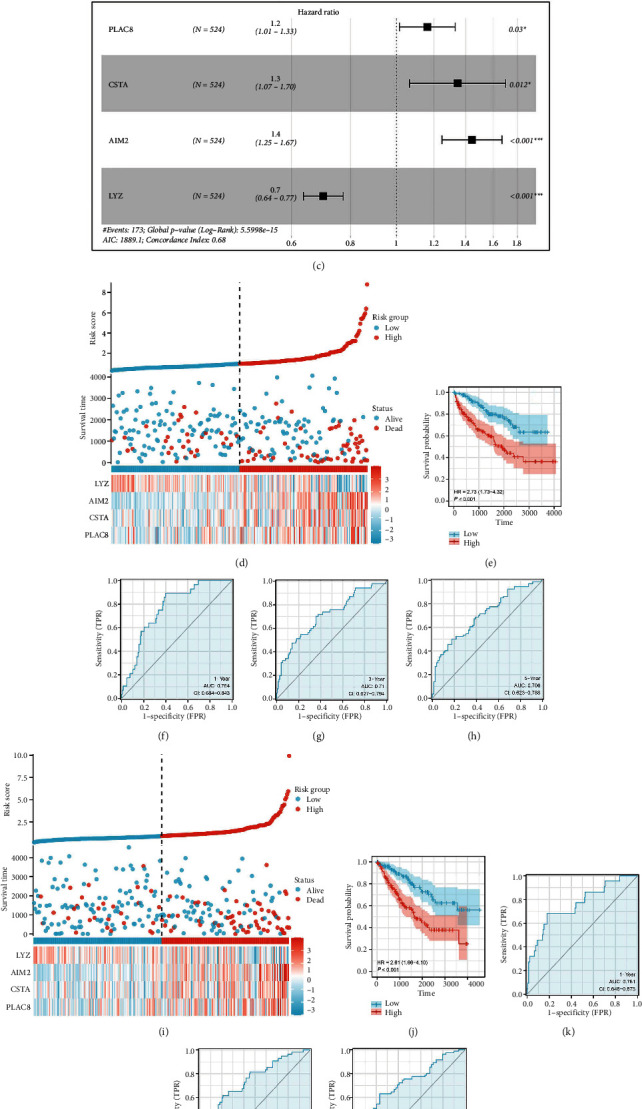
Prognosis model. (a) and (b) LASSO regression analysis; (c) multivariate Cox regression analysis for model construction; (d) overview of prognosis model in the training cohort; (e) KM survival between high- and low-risk patients (training cohort); (f)–(h) ROC curves of 1-, 3-, and 5-year survival of our model in the training cohort; (i) overview of prognosis model in the validation cohort; (j) KM survival between high- and low-risk patients (validation cohort); (k)–(m) ROC curves of 1-, 3-, and 5-year survival of our model in the validation cohort.

We then evaluated the expression pattern of the 55 rejection-related genes in cancers. Interestingly, the results revealed that all of these genes were upregulated in ccRCC patients (Kidney renal clear cell carcinoma (KIRC) project) (Figure 2(a)). The ClueGO analysis showed that these rejection-related genes were primarily enriched in the cellular response to interferon-gamma, natural killer cells medicated immunity, interleukin-12 production, neuroinflammatory response, positive regulation of innate immune response, antimicrobial humoral immune response mediated by antimicrobial peptide, chronic inflammatory response, regulation of T cell proliferation, lymphocyte chemotaxis, and neutrophil chemotaxis (Figure 2(b)). The ccRCC patients in TCGA database were randomly assigned to the training and validation group in a ratio of 1 : 1. The univariate Cox regression analysis indicated that among the rejection-related genes, 14 were significantly correlated with ccRCC patients' survival (Figures 2(c) and 2(d), *P* < 0.05). Subsequently, the LASSO regression analysis was used to screen the most significant variables (Figures 3(a) and 3(b)). Based on the identified genes, multivariate Cox regression analysis was used to establish a prognosis model consisting of four genes, *PLAC8*, *CSTA*, *AIM2*, and *LYZ* (Figures 3(c) and 3(d)). According to the Kaplan–Meier survival curve, patients in the high-risk group showed a poorer survival rate than those in the low-risk group (Figure 3(e)). Furthermore, the Receiver Operating Characteristic (ROC) curve showed a good performance for survival prediction at 1, 3, and 5 years (Figures 3(f), 3(g), and 3(h); 1-year AUC = 0.764, 3-year AUC = 0.71, 5-year AUC = 0.706). Similarly, the validation cohort showed the same pattern (Figures 3(i), 3(j), 3(k), 3(l), and 3(m)).

### 3.3. Machine Learning Algorithms Identify the Rejection-Related Genes Involved in Cancer Occurrence

**Figure 4:**
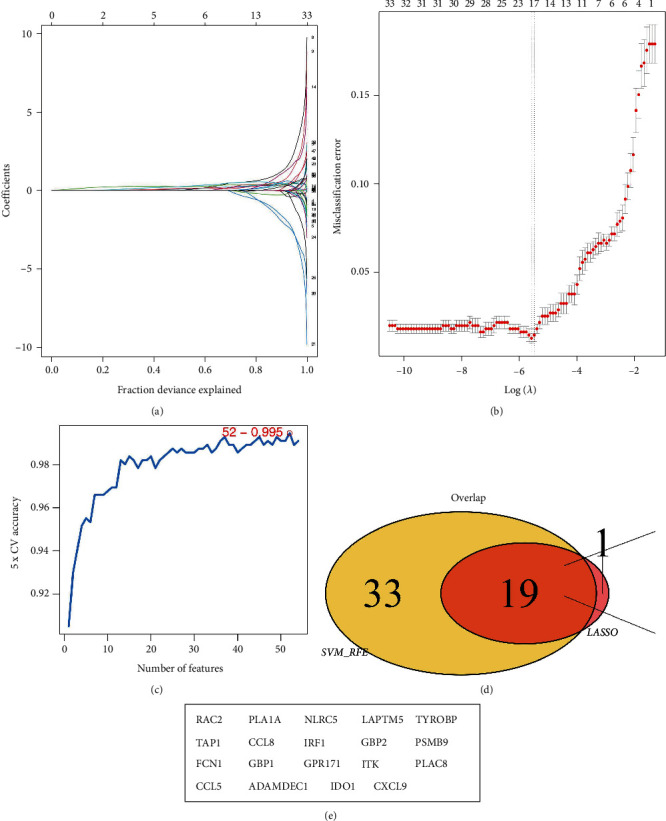
Machine learning algorithms identify the rejection-related genes involved in cancer occurrence. (a) and (b) LASSO logistics regression algorithm; (c) SVM-RFE algorithm; (d) and (e) machine learning algorithms identified 19 rejection-related genes involved in ccRCC occurrence.

**Figure 5:**
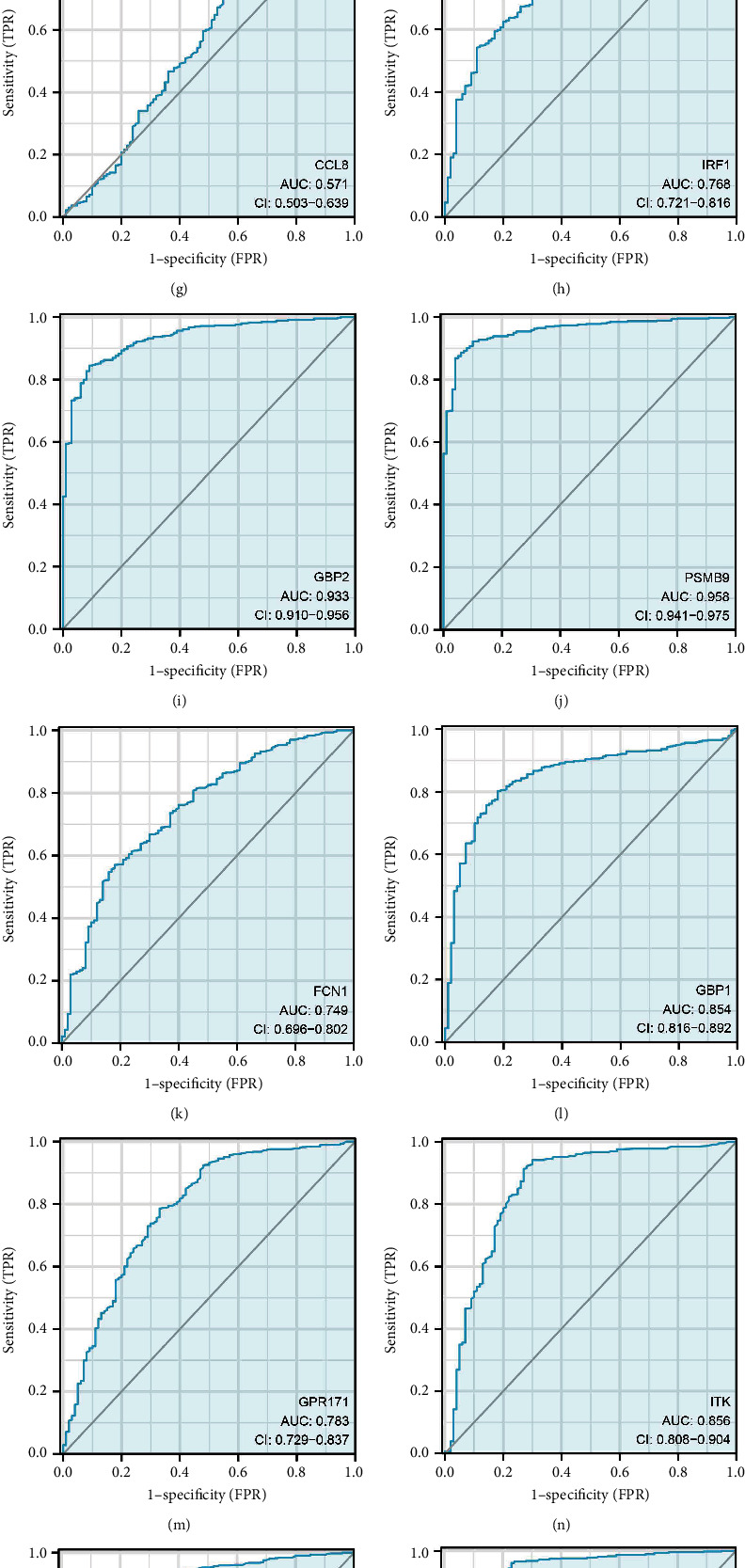
Prediction efficiency of 19 rejection-related genes in ccRCC diagnosis. (a) ROC curves of RAC2; (b) ROC curves of PLA1A; (c) ROC curves of NLRC5; (d) ROC curves of LAPTM5; (e) ROC curves of TYROBP; (f) ROC curves of TAP1; (g) ROC curves of CCL8; (h) ROC curves of IRF1; (i) ROC curves of GBP2; (j) ROC curves of PSMB9; (k) ROC curves of FCN1; (l) ROC curves of GBP1; (m) ROC curves of GRP171; (n) ROC curves of ITK; (o) ROC curves of PLAC8; (p) ROC curves of CCL5; (q) ROC curves of ADAMDEC1; (r) ROC curves of IDO1; (s) ROC curves of CXCL9; (t) ROC curves of the combined score.

The LASSO logistics regression and SVM-RFE algorithms (Figures 4(a), 4(b), and 4(c)) revealed that 19 rejection-related genes involved in ccRCC occurrence, including *RAC2*, *PLA1A*, *NLRC5*, *LAPTM5*, *TYROBP*, *TAP1*, *CCL8*, *IRF1*, *GBP2*, *PSMB9*, *FCN1*, *GBP1*, *GPR171*, *ITK*, *PLAC8*, *CCL5*, *ADAMDEC1*, *IDO1*, and *CXCL9* (Figures 4(d) and 4(e)). The ROC curves showed that these genes had a good diagnosis efficiency for ccRCC diagnosis (Figures 5(a), 5(b), 5(c), 5(d), 5(e), 5(f), 5(g), 5(h), 5(i), 5(j), 5(k), 5(l), 5(m), 5(n), 5(o), 5(p), 5(q), 5(r), 5(s), and 5(t)).

### 3.4. Further Exploration of PLAC8 in ccRCC

**Figure 6:**
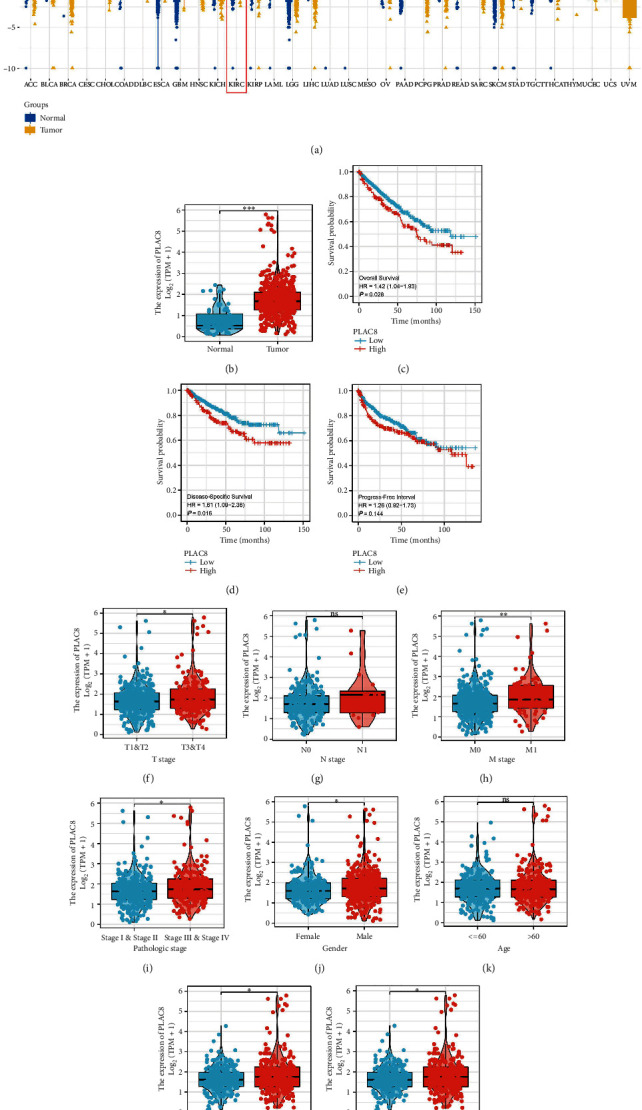
Clinical role of PLAC8 in ccRCC. (a) Expression pattern of PLAC8 in pan-cancer; (b) expression level of PLAC8 in ccRCC and control tissue; (c)–(e) PLCA8 level tends to have a worse overall survival, disease-free survival, and progression-free survival; (f)–(m) expression level of PLAC8 in patients with different groups.

**Figure 7:**
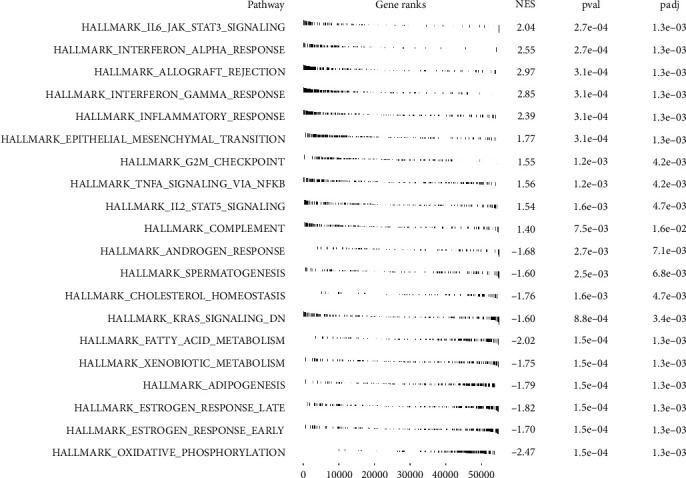
Biological enrichment analysis of PLAC8 in ccRCC.

Only the *PLAC8* gene was significantly associated with patients' prognosis (multivariate Cox regression) and was involved in the occurrence of ccRCC. Therefore, we selected it for further analysis. The pan-cancer analysis showed that *PLCA8* was differentially expressed in various cancer types, including ccRCC (Figures 6(a) and 6(b)). Furthermore, the Kaplan–Meier survival curves revealed that the patients with a high expression of *PLCA8* had poorer overall survival, disease-free survival, and progression-free survival than those with a low expression of *PLAC8* (Figures 6(c), 6(d), and 6(e)). Furthermore, the clinical correlation analysis showed a higher expression of PLCA8 in the T3-4 patients, M1 patients, Stage III–IV patients, male patients, and G3–4 patients than the control group (Figures 6(f), 6(g), 6(h), 6(i), 6(j), 6(k), 6(l), and 6(m)). Moreover, the GSEA analysis showed a high expression of *PLCA8* in the interleukin 6 (IL6)/Janus Kinase (JAK)/signal transducer and activator of transcription 3 (STAT3) signaling pathway, interferon-alpha response, allograft rejection, interferon-gamma response, and epithelial–mesenchymal transition (Figure 7).

## 4. Discussion

ccRCC is the most prevalent subtype of RCC and is a significant public health challenge [23]. According to previous studies, kidney transplantation recipients (KTRs) exposed to immunosuppression for more than 20 years were more likely to suffer from RCC [6, 24]. However, the specific mechanisms leading to the occurrence and development of ccRCC after transplantation are still unknown. In this study, we employed machine learning algorithms to identify the genes associated with renal cancer. Machine learning is a powerful tool in radiogenomics as it allows the integration of imaging and genomics data [25, 26]. Furthermore, machine learning could provide valuable insights into ccRCC due to the relative lack of mutant genes. With the help of machine learning, combining target gene detection with radiogenomics offers an opportunity for accurate diagnosis, prognosis, and treatment option determination [27].

*PLAC8*, also known as onzin, was initially identified in mid-gestation placentas and mice embryos using genome-wide expression profiling [28, 29]. In addition, PLAC8 has been identified in human cells such as plasmacytoid dendritic cells [30], lymphoid cells, myeloid cells, and intestinal epithelial cells [31]. According to previous studies, PLAC8 is a cysteine-rich protein that plays crucial roles in cell proliferation, cell immunity, cell apoptosis, and cancer pathophysiology [32–35]. Although the precise role of *PLAC8* in tumorigenesis remains unclear, recent studies have shown that *PLAC8* plays multiple roles across various cell types. For example, PLAC8 induces epithelial–mesenchymal transition in colon carcinoma cells [36]; regulates PD-L1 ubiquitination levels in breast cancer cells, thus influencing immune response and cancer cell proliferation [37]; and triggers oncogenic autophagy, thus affecting autophagosome–lysosome fusion in pancreatic cells [38].

The present study showed that *PLAC8* could affect tumorigenesis in ccRCC by regulating the IL6/JAK/STAT3 signaling pathway, allograft rejection, interferon-alpha response, epithelial–mesenchymal transition, and interferon-gamma response. Consistent with our findings, Shi et al. reported that PLAC8 affects tumorigenesis by regulating immunity and inflammatory processes [39]. In addition, the present study revealed that the expression of PLAC8 could be used to predict clinical outcomes in ccRCC [39]. Also, previous studies showed that *PLAC8* may be a biomarker of epithelial mesenchymal transitions progression and cancer metastasis [40].

The IL6–JAK–STAT3 signaling pathway plays diverse roles in tumorigenesis, including angiogenesis, tumor invasion, and migration [41–44]. Zhan et al. demonstrated that a prognosis model based on the IL6–JAK–STAT3 pathway-related genes had a good predictive performance for diagnosing ccRCC [45].

The incidence of RCC in KTRs remains unclear. Furthermore, the development of RCC in KTRs could be an interplay of various factors. Firstly, long-term therapy with immunosuppressive drugs is associated with reduced immune surveillance, resulting in decreased ability to detect and clear abnormal cells, including tumor cells [46]. Secondly, the immunosuppressive drugs used in renal transplant patients could have tumorigenesis effects [46]. Furthermore, long-term dialysis treatment increases the risk of developing renal carcinoma. Moreover, patients with ESRD on prolonged dialysis have a higher risk of developing acquired cystic nephropathy, which, in turn, increases the risk of developing renal cancer [47]. The occurrence of kidney transplant rejection is often complex and may be related to multiple cells or antibodies, such as antibody-mediated rejection (ABMR), T cell-mediated rejection (TCMR), and other types of rejection [48]. Among them, TCMR and ABMR are the most important types. Although the precise mechanism behind ABMR remains elusive, researchers generally believe that its occurrence is related to the interaction of donor-specific alloantibodies (DSAs) against donor human leukocyte antigen antigens [49]. Persistent T-cell damage can lead to the occurrence of TCMR [50]. Different subtypes often have different pathological and physiological processes, which poses challenges for the diagnosis and treatment of Ktx [51].

There were several limitations to our research. Firstly, the data analyzed in this study originated from Western countries. Therefore, the findings of this study might not apply to patients in Asian patients. Secondly, this study had a limited sample size. Thirdly, this study did not investigate the role of other identified genes in the progression of ccRCC in Ktx. Therefore, further studies are needed to investigate the role of the other genes. Fourthly, the credibility of our findings could be undermined by the lack of clinical data. Fifthly, we only evaluate the prognosis value of rejection-related genes in TCGA cohort. For example, the prognostic value of PLAC8 in other different ccRCC cohorts still cannot be effectively validated. Therefore, our results can only provide directional significance and still need to be re-evaluated when applied to new cohorts. Lastly, we did not focus on different types of rejection. The rejection group in our study includes all types of renal rejection, including ABMR, TCMR, and other kinds of rejection. The potential biological differences between different subtypes can to some extent reduce the credibility of our conclusions, especially when focusing on a specific rejection subtype.

## 5. Conclusion

This study identified the molecules involved in the rejection of renal transplant patients. PLAC8, a rejection-related gene, was found associated with ccRCC prognosis and occurrence, which might be a potential target. This study provides novel insights into post-renal transplant research in patients with ccRCC.

## Figures and Tables

**Table 1:** The baseline information of ccRCC patients.

Clinical features		Number	Percentage (%)
Age	≤65	352	65.5
	>65	185	34.5
Gender	Female	191	35.6
	Male	346	64.4
Grade	G1	14	2.6
	G2	230	42.8
	G3	207	38.5
	G4	78	14.5
	Unknown	8	1.5
Stage	Stage I	269	50.1
	Stage II	57	10.6
	Stage III	125	23.3
	Stage IV	83	15.5
	Unknown	3	0.6
T stage	T1	275	51.2
	T2	69	12.8
	T3	182	33.9
	T4	11	2.1
M stage	M0	426	79.3
	M1	79	14.7
	Unknown	32	6.0
N stage	N0	240	44.7
	N1	17	3.2
	Unknown	280	52.1

**Table 2:** The baseline information of participants from GEO databases.

Dataset	Platform	Sample size	Tissue source	Histologic diagnosis (numbers)
GSE48581	GPL570	306	Renal allograft biopsy	ABMR (32), mixed (4), TCMR (29), others (151), NA (48), borderline (42)
GSE36059	GPL570	411	Renal allograft biopsy	ABMR (51), mixed (13), TCMR (20), others (188), NA (107), borderline (32)
GSE98320	GPL15207	1207	Renal allograft biopsy	ABMR (239), AKI (96), BK (37), Bord (109), DiabNeph (18), GN (97), IFTA (145), mixed (41), NOMOA (274), other (25), TCMR (87), TG (40)

## Data Availability

The transcriptional profile data of kidney transplant patients with or without rejection response can be obtained from GSE48581, GSE36059, and GSE98320 in Gene Expression Omnibus (GEO) database (https://www.ncbi.nlm.nih.gov/geo/). Pan-cancer data was obtained from the USCS XENA website (https://xenabrowser.net/).

## Supplementary Materials

Supplementary Materials Figure S1. The flow chart of whole study.

## References

[1] Shuch B., Amin A., Armstrong A. J. (2015). Understanding pathologic variants of renal cell carcinoma: distilling therapeutic opportunities from biologic complexity. *European Urology*.

[2] Capitanio U., Montorsi F. (2016). Renal cancer. *Lancet*.

[3] Xu J., Reznik E., Lee H. J. (2019). Abnormal oxidative metabolism in a quiet genomic background underlies clear cell papillary renal cell carcinoma. *eLife*.

[4] Escudier B., Porta C., Schmidinger M. (2016). Renal cell carcinoma: ESMO Clinical Practice Guidelines for diagnosis, treatment and follow-up^†^. *Annals of Oncology*.

[5] Hariharan S., Johnson C. P., Bresnahan B. A., Taranto S. E., McIntosh M. J., Stablein D. (2000). Improved graft survival after renal transplantation in the United States, 1988 to 1996. *The New England Journal of Medicine*.

[6] Fuhrmann J. D., Valkova K., von Moos S., Wuthrich R. P., Muller T. F., Schachtner T. (2022). Cancer among kidney transplant recipients >20 years after transplantation: post-transplant lymphoproliferative disorder remains the most common cancer type in the ultra long-term. *Clinical Kidney Journal*.

[7] Chewcharat A., Thongprayoon C., Bathini T. (2019). Incidence and mortality of renal cell carcinoma after kidney transplantation: a meta-analysis. *Journal of Clinical Medicine*.

[8] Braconnier P., Del Marmol V., Broeders N. (2012). Combined introduction of anti-IL2 receptor antibodies, mycophenolic acid and tacrolimus: effect on malignancies after renal transplantation in a single-centre retrospective cohort study. *Nephrology, Dialysis, Transplantation*.

[9] Dantal J., Hourmant M., Cantarovich D. (1998). Effect of long-term immunosuppression in kidney-graft recipients on cancer incidence: randomised comparison of two cyclosporin regimens. *Lancet*.

[10] Farrugia D., Mahboob S., Cheshire J. (2014). Malignancy-related mortality following kidney transplantation is common. *Kidney International*.

[11] Zhang X., Ren X., Zhang T. (2022). Comprehensive analysis of the association between human non-obstructive azoospermia and plasticisers via single-cell and traditional RNA sequencing methods.

[12] Zhang X., Ren X., Zhang T. (2022). Comprehensive analysis of the association between human non-obstructive azoospermia and plasticisers via single-cell and traditional RNA sequencing methods. *Exposure and Health*.

[13] Zhang T., Zhou X., Zhang X. (2022). Gut microbiota may contribute to the postnatal male reproductive abnormalities induced by prenatal dibutyl phthalate exposure. *Chemosphere*.

[14] Ren X., Zhang T., Chen X. (2020). Early-life exposure to bisphenol A and reproductive-related outcomes in rodent models: a systematic review and meta-analysis. *Aging*.

[15] Yuan M., Hu X., Yao L., Liu P., Jiang Y., Li L. (2022). Comprehensive bioinformatics and machine learning analysis identify VCAN as a novel biomarker of hepatitis B virus-related liver fibrosis. *Frontiers in Molecular Biosciences*.

[16] Ritchie M. E., Phipson B., Wu D. (2015). Limma powers differential expression analyses for RNA-sequencing and microarray studies. *Nucleic Acids Research*.

[17] von Mering C., Huynen M., Jaeggi D., Schmidt S., Bork P., Snel B. (2003). STRING: a database of predicted functional associations between proteins. *Nucleic Acids Research*.

[18] Subramanian A., Tamayo P., Mootha V. K. (2005). Gene set enrichment analysis: a knowledge-based approach for interpreting genome-wide expression profiles. *Proceedings of the National Academy of Sciences of the United States of America*.

[19] Bindea G., Mlecnik B., Hackl H. (2009). ClueGO: a Cytoscape plug-in to decipher functionally grouped gene ontology and pathway annotation networks. *Bioinformatics*.

[20] Deo R. C. (2015). Machine learning in medicine. *Circulation*.

[21] Huang M. L., Hung Y. H., Lee W. M., Li R. K., Jiang B. R. (2014). SVM-RFE based feature selection and Taguchi parameters optimization for multiclass SVM classifier. *The Scientific World Journal*.

[22] Tang J., Mou M., Wang Y., Luo Y., Zhu F. (2021). MetaFS: performance assessment of biomarker discovery in metaproteomics. *Briefings in Bioinformatics*.

[23] Rini B. I., Campbell S. C., Escudier B. (2009). Renal cell carcinoma. *Lancet*.

[24] Collett D., Mumford L., Banner N. R., Neuberger J., Watson C. (2010). Comparison of the incidence of malignancy in recipients of different types of organ: a UK registry audit. *American Journal of Transplantation*.

[25] Ferro M., Musi G., Marchioni M. (2023). Radiogenomics in renal cancer management-current evidence and future prospects. *International Journal of Molecular Sciences*.

[26] Alessandrino F., Shinagare A. B., Bosse D., Choueiri T. K., Krajewski K. M. (2019). Radiogenomics in renal cell carcinoma. *Abdominal Radiology*.

[27] Tataru O. S., Marchioni M., Crocetto F. (2023). Molecular imaging diagnosis of renal cancer using 99mTc-Sestamibi SPECT/CT and Girentuximab PET-CT-current evidence and future development of novel techniques. *Diagnostics*.

[28] Galaviz-Hernandez C., Stagg C., de Ridder G. (2003). Plac8 and Plac9, novel placental-enriched genes identified through microarray analysis. *Gene*.

[29] Tanaka T. S., Jaradat S. A., Lim M. K. (2000). Genome-wide expression profiling of mid-gestation placenta and embryo using a 15,000 mouse developmental cDNA microarray. *Proceedings of the National Academy of Sciences of the United States of America*.

[30] Rissoan M. C., Duhen T., Bridon J. M. (2002). Subtractive hybridization reveals the expression of immunoglobulin-like transcript 7, Eph-B1, granzyme B, and 3 novel transcripts in human plasmacytoid dendritic cells. *Blood*.

[31] Ledford J. G., Kovarova M., Koller B. H. (2007). Impaired host defense in mice lacking ONZIN. *Journal of Immunology*.

[32] Jimenez-Preitner M., Berney X., Thorens B. (2012). Plac8 is required for white adipocyte differentiation in vitro and cell number control in vivo. *PLoS One*.

[33] Kinsey C., Balakrishnan V., O’Dell M. R. (2014). Plac8 links oncogenic mutations to regulation of autophagy and is critical to pancreatic cancer progression. *Cell Reports*.

[34] Mourtada-Maarabouni M., Watson D., Munir M., Farzaneh F., Williams G. T. (2013). Apoptosis suppression by candidate oncogene PLAC8 is reversed in other cell types. *Current Cancer Drug Targets*.

[35] Segawa S., Kondo Y., Nakai Y. (2018). Placenta specific 8 suppresses IL-18 production through regulation of autophagy and is associated with adult still disease. *Journal of Immunology*.

[36] Li C., Ma H., Wang Y. (2014). Excess PLAC8 promotes an unconventional ERK2-dependent EMT in colon cancer. *The Journal of Clinical Investigation*.

[37] Mao M., Chen Y., Yang J. (2022). Modification of PLAC8 by UFM1 affects tumorous proliferation and immune response by impacting PD-L1 levels in triple-negative breast cancer. *Journal for Immunotherapy of Cancer*.

[38] Kaistha B. P., Lorenz H., Schmidt H. (2016). PLAC8 localizes to the inner plasma membrane of pancreatic cancer cells and regulates cell growth and disease progression through critical cell-cycle regulatory pathways. *Cancer Research*.

[39] Shi L., Xiao L., Heng B., Mo S., Chen W., Su Z. (2017). Overexpression of placenta specific 8 is associated with malignant progression and poor prognosis of clear cell renal cell carcinoma. *International Urology and Nephrology*.

[40] Mao M., Cheng Y., Yang J. (2021). Multifaced roles of PLAC8 in cancer. *Biomarker Research*.

[41] Ni J. S., Zheng H., Ou Y. L. (2020). miR-515-5p suppresses HCC migration and invasion via targeting IL6/JAK/STAT3 pathway. *Surgical Oncology*.

[42] Pan M. S., Wang H., Ansari K. H., Li X. P., Sun W., Fan Y. Z. (2021). Correction to: gallbladder cancer-associated fibroblasts promote vasculogenic mimicry formation and tumor growth in gallbladder cancer via upregulating the expression of NOX4, a poor prognosis factor, through IL-6-JAK-STAT3 signal pathway. *Journal of Experimental and Clinical Cancer Research*.

[43] Siersbaek R., Scabia V., Nagarajan S. (2020). IL6/STAT3 signaling hijacks estrogen receptor *α* enhancers to drive breast cancer metastasis. *Cancer Cell*.

[44] Tung K. L., Wu Y. T., Liu C. (2020). EBV Rta-induced IL-6 promotes migration of bystander tumor cells through IL-6R/JAK/STAT3 pathway in vitro. *Anticancer Research*.

[45] Zhan C., Xu C., Chen J. (2021). Development and validation of an IL6/JAK/STAT3-related gene signature to predict overall survival in clear cell renal cell carcinoma. *Frontiers in Cell and Development Biology*.

[46] Eggers H., Guler F., Ehlers U., Ivanyi P., Peters I., Grunwald V. (2019). Renal cell carcinoma in kidney transplant recipients: descriptive analysis and overview of a major German transplant center. *Future Oncology*.

[47] Hoshida Y., Nakanishi H., Shin M., Satoh T., Hanai J., Aozasa K. (1999). Renal neoplasias in patients receiving dialysis and renal transplantation: clinico-pathological features and p53 gene mutations. *Transplantation*.

[48] Lai X., Zheng X., Mathew J. M., Gallon L., Leventhal J. R., Zhang Z. J. (2021). Tackling chronic kidney transplant rejection: challenges and promises. *Frontiers in Immunology*.

[49] Crespo M., Llinàs-Mallol L., Redondo-Pachón D. (2021). Non-HLA antibodies and epitope mismatches in kidney transplant recipients with histological antibody-mediated rejection. *Frontiers in Immunology*.

[50] Salem F., Perin L., Sedrakyan S. (2022). The spatially resolved transcriptional profile of acute T cell-mediated rejection in a kidney allograft. *Kidney International*.

[51] Loupy A., Haas M., Roufosse C. (2020). The Banff 2019 kidney meeting report (I): updates on and clarification of criteria for T cell- and antibody-mediated rejection. *American Journal of Transplantation*.

