# Supplementary figures and images for: Investigation of Underlying Biological Association and Targets between Rejection of Renal Transplant and Renal Cancer
Source: Int J Genomics. 2023 May 23;2023:5542233. doi: 10.1155/2023/5542233 (PMC10229252; doi:10.1155/2023/5542233)

**Supplementary file**  
**Figure S1. The flow chart of whole study**

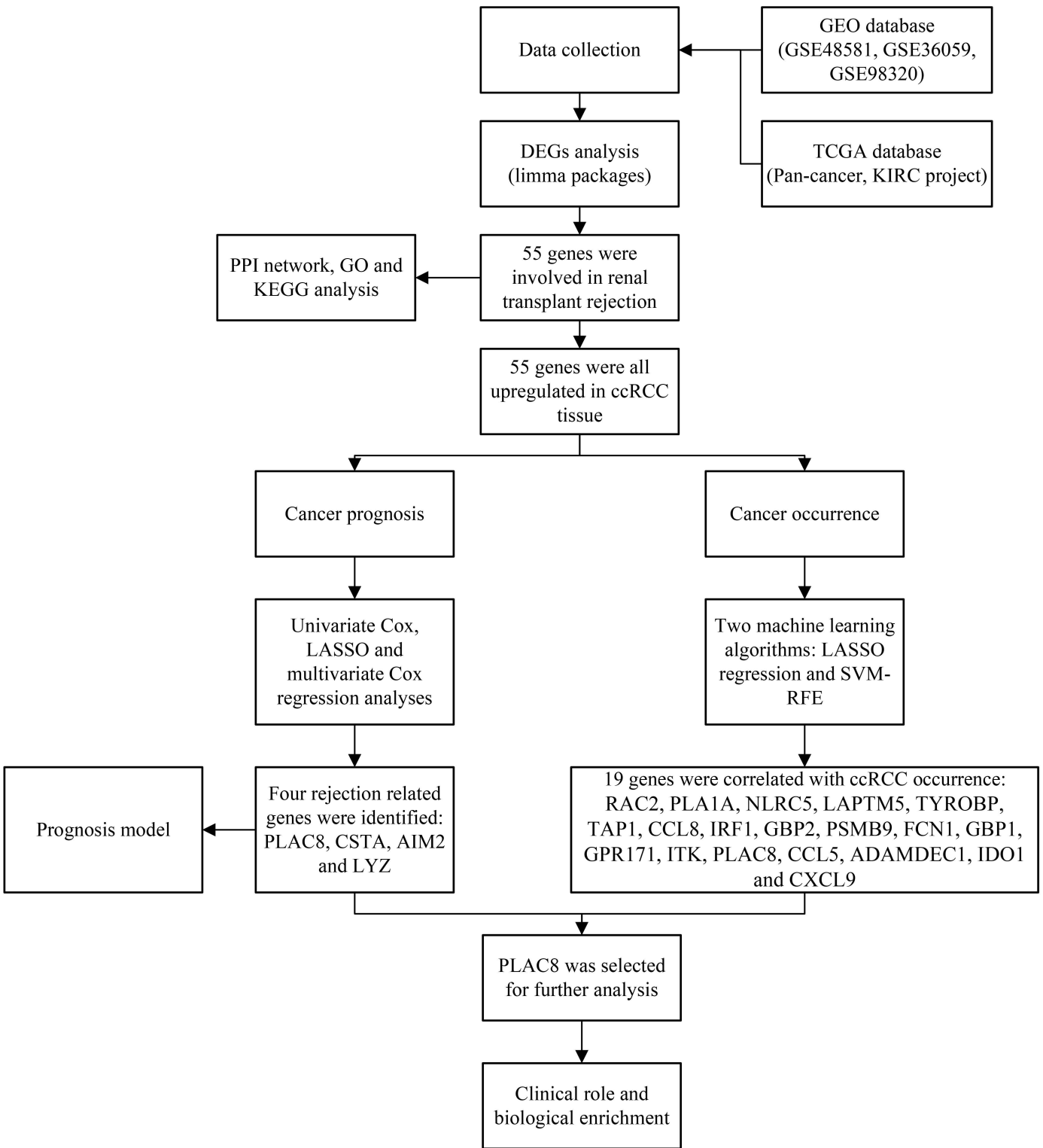

Supplement: Supplementary Materials — Figure S1. The flow chart of whole study. [file 5542233.f1.pdf]
